# Repeated primary care consultations for non-specific physical symptoms in children in UK: a cohort study

**DOI:** 10.1186/s12875-014-0195-4

**Published:** 2014-12-05

**Authors:** Mujahed Shraim, Milisa Blagojevic-Bucknall, Christian D Mallen, Kate M Dunn

**Affiliations:** Arthritis Research UK Primary Care Centre, Keele University, Keele, UK; Work Environment Department, University of Massachusetts Lowell, Lowell, Massachusetts USA; Center for Disability Research, Liberty Mutual Research Institute for Safety, Hopkinton, Massachusetts USA

## Abstract

**Background:**

Non-specific physical symptoms (NSPS), such as headache and abdominal pain, are common reasons for children to consult primary care. NSPS represent a significant burden not only on society, but also on health care services, through frequent physician consultations and referrals to secondary care. Research evidence suggests a positive relationship between health and consulting behavior of parents and their children, but research on whether repeated physician consultations for NSPS in children is influenced by parental consultations for NSPS is lacking. The aim was to measure the frequency of repeated physician consultations for NSPS in children, and investigate whether this is influenced by maternal consultations for NSPS.

**Methods:**

A cohort study of children registered with primary care practices contributing to the Consultation in Primary Care Archive database. Participants were child-mother pairs registered between January 2007 and December 2010. The cohort comprised all children (n = 1437) aged 2 to 16 years who consulted a physician for NSPS in 2009. Mothers’ consultations for NSPS were measured between 2007 and 2008. Main outcome measures were repetition and frequency of consultations for NSPS in children (consultations for NSPS in both 2009 and 2010).

**Results:**

Overall, 27% of children had repeated consultations for NSPS. The three most common repeated consultations were for back pain, constipation and abdominal pain. Exposure to maternal consultation for NSPS was associated with 21% increase in consultation frequency for NSPS (adjusted incidence rate ratio 1.21; 95% CI 1.12, 1.31). After adjusting for child age and maternal age, maternal consultation for NSPS was associated with an increased risk of repeated consultations for NSPS in children (relative risk 1.41; 95% CI 1.16, 1.73). This association was also significant for specific NSPS groups including painful, gastrointestinal, and neurologic symptoms.

**Conclusions:**

Repeated consultation for NSPS is common among children. It is important for primary care physicians and secondary care clinicians, managing children referred from primary care for NSPS, to be aware that consultation for NSPS in mothers is a risk factor for repeated consultations for NSPS among children. More research is needed to uncover exactly how parental health influences health and consulting behavior of children.

## Background

Non-specific physical symptoms (NSPS) are defined as physical symptoms that lead patients to seek healthcare, and after clinical examination are not explained by clear pathological changes [1]. NSPS, such as musculoskeletal pain, abdominal pain and headache are common in children [2,3]. Annually, one third of children consult a physician for NSPS [4,5]. NSPS among children are associated with functional impairment and negative impact on quality of life of children and their parents [6,7]. This represents a significant burden on healthcare services through frequent consultations, diagnostic testing, and secondary care referrals [8-10]. The causes of NSPS in children are yet to be fully explained, but are likely to be multifactorial, including genetic and psychosocial factors, including parental influence on childhood illness and health-seeking behavior [11-13]. A recent systematic review found evidence of an association between physician consultations for NSPS in parents and children, but its findings were limited by the methodology of empirical studies, including cross-sectional designs, reliance on self-reported data, and including children from specific age groups only [14].

Population-based studies have demonstrated that NSPS persist in many children over time [15-17]. Additionally, a recent study reported an association between parental history of functional pain syndromes, such as migraine and recurrent abdominal pain, and chronic pain in their children [18]. However, evidence that this translates to physician consultations for NSPS is lacking. It is unknown whether repeated consultation for NSPS in children is influenced by parental physician consultations for NSPS. Information about the likely outcomes of children consulting physicians for NSPS is important in order to improve the quality of healthcare and patient outcomes [19].

The objective of this study was to quantify the frequency of repeated physician consultations for NSPS in children, including different patterns of NSPS, and investigate whether frequency and repetition of consultation for NSPS in children are influenced by maternal consultations for NSPS.

## Methods

### Study design and setting

This was a cohort study of children registered with primary care practices. We used the Consultation in Primary Care Archive (CiPCA), a primary care research database containing consultations occurring at 12 practices in North Staffordshire, UK. The total population registered mid-year 2009 was 104,911. CiPCA is a high-quality, anonymized and validated database, the contributing practices have regular cycles of training, assessment and feedback with respect to the quality of coded clinical data [20]. Data from CiPCA on the annual consultation prevalence for musculoskeletal conditions are comparable to data from larger national primary care databases [21].

Ethical approval for the CiPCA database was given by the North Staffordshire and Staffordshire Research Ethics Committees (UK), who gave permission to download and store anonymized medical record information for research use from participating general practices. All general practices participating in CiPCA inform their patient populations that their anonymized records will be used in this way and all patients are offered the opportunity to withdraw their records from inclusion in CiPCA.

### Participants

Eligible participants were children and mothers registered with CiPCA practices between January 2007 and December 2010. The cohort consisted of all children aged 2 to 16 years in 2009 who consulted a physician for NSPS in 2009. One child per household was randomly selected because the main exposure of interest was maternal physician consultation for NSPS, which would be the same for siblings, thus avoiding over representing families with more than one child. About 99.3% of babies born in England and Wales in 2010 have a mother aged 17 to 45 years at the birth of the child [22]. Therefore, a mother was defined as a female aged between 17 and 45 years at the birth of the child and bearing the same household identification code (to identify all persons living in a household and registered with the same practice). We excluded households with more than one female meeting our definition of a mother to minimize the potential for classifying sisters or grandmothers as mothers of selected children.

### Outcome measures

The primary outcome measure was repetition of consultation for NSPS in children, dichotomized as: (a) children with repeated consultations for NSPS, consisting of children who consulted for NSPS in 2009 and at least once in 2010; (b) children without repeated consultations for NSPS, consisting of children who consulted for NSPS in 2009 but not in 2010. We also measured the frequency of consultation for NSPS among children in 2009 and 2010.

NSPS consultations were defined using a comprehensive list of standardized diagnostic Read codes for physical symptoms (presented in Table 1). Read codes are a hierarchy of morbidity, symptoms and process codes that are used to record all electronic morbidity data in UK primary care [23]. The full list of Read codes for physical symptoms is available from the authors.

**Table 1:** **List of non-specific physical symptoms**

**Non-specific physical symptoms according to bodily system**	
**Musculoskeletal symptoms**	Burning sensation in sexual organs or rectum
Pain in extremities	Dysmenorrhea (painful menstruation)
Back pain	Metrorrhagia (irregular menstrual periods)
Joint pain	Menorrhagia (heavy menstrual bleeding)
Muscles soreness	Sexual indifference (decreased libido)*
**Gastrointestinal symptoms**	Dyspareunia (pain during intercourse)*
Vomiting	**Neurologic symptoms**
Abdominal pain	Dizziness
Nausea	Fainting (syncope) or loss of consciousness
Abdominal bloating	Transient Amnesia (loss of memory)
Diarrhea	Transient Aphonia (loss of voice)
Constipation	Transient Diplopia (double vision)
Multiple food intolerance	Transient blurred vision
Globus (lump in the throat)	Transient blindness
Dysphagia (difficulty swallowing)	Transient seizure or convulsion
**Cardiopulmonary symptoms**	Transient Ataxia (trouble walking)
Palpitations	Transient Paresis (paralysis)
Chest pain	Paresthesia (numbness or tingling sensation)
Hyperventilation or Dyspnea	Headache
Hot or cold spells (sweat)	Weakness in parts of the body
**Urogenital symptoms**	Heavy feelings in arms or legs
Pain during urination	**General symptoms**
Difficulty urinating (Dysuria)	Fatigue

*Symptoms were excluded from analysis for children.

NSPS are usually recorded and coded in the physician’s computer system as symptom diagnoses when a precise diagnosis is unavailable. We classified consultations as NSPS if physical symptoms diagnoses were coded as the main reason for encounter, and the free-text records suggested that the cause was not-specific (e.g. a girl consulting with headache without objective pathological changes on physical examination and/or diagnostic testing). We excluded consultations for physical symptoms due to trauma or injury. We also excluded consultations for included physical symptoms in pregnant mothers at the time of consultations.

We examined the repetition of consultation for NSPS in children by type of NSPS (painful and not-painful symptoms) and by three body systems (musculoskeletal (e.g. joint pain), gastrointestinal (e.g. abdominal pain), and neurological symptoms (e.g. headache)); see Table 1.

### Ascertainment of maternal consultations for NSPS

All consultations made by mothers between 1 January 2007 and 31 December 2008 were extracted. Consultations for NSPS in mothers were identified and classified into groups using the same method described for children. The child exposure to maternal consultation for NSPS was defined as exposure to at least one maternal consultation for NSPS between 1 January 2007 and 31 December 2008.

### Measurement of other variables

We extracted data on sociodemographic and health related characteristics for children and mothers from the CiPCA database. Sociodemographic variables included child sex and age, child birth order, household members’ count, number of siblings in household, index of multiple deprivation (IMD) 2007 scores for residential area level deprivation, and maternal age. Health related variables included primary care practice and maternal history of anxiety or depressive disorders.

Children’s age was split into tertiles (2-6, 7-11 and 12-16 years), and maternal age into quartiles (19-28, 29-39, 40-50 and 51-61 years). Maternal age in 2009 was used to reflect their age at the time of children’s consultation.

The household identification codes were used to identify all household members of included children. Younger siblings for index children were defined as persons from the same household born after the index child, whereas older siblings were defined as persons from the same household and aged 16 or less at the birth of the index child. The birth order of the child was classified as “first” if the child had no siblings or if the child was the oldest child in the household (with no other household members’ meeting the definition for a sibling). Households with 13 or more members were excluded to prevent including families living in shared households.

The IMD 2007 scores were constructed by the Department of Communities and Local Government, and conceptualized as a weighted area level aggregation of scores for seven domains of deprivation including income; employment; health deprivation and disability; education, skills and training; barriers to housing and services; crime; living environment [24]. IMD 2007 scores range from 0% to 100% where higher scores indicate greater deprivation [24]. The IMD 2007 scores for children’s residential area level deprivation were presented as quintiles with ‘1’ representing the most affluent and ‘5’ presenting the most deprived.

Maternal history of anxiety/depressive disorders was identified by searching mothers’ records between 2007 and 2008 using a list of pre-defined anxiety/depression Read codes (available on request from the authors).

### Statistical analysis

Chi-squared tests and Mann-Whitney U tests were performed to test for significant baseline differences between exposed and unexposed children to maternal NSPS consultation. The Cox proportional hazards regression with invariant time was used to obtain estimates of relative risk (RR) and associated 95% confidence intervals (CI) as a summary measure of association between predictors and repetition of consultation for NSPS in children. Univariable analyses were initially performed to obtain individual associations between each predictor and repeated consultation for NSPS. All significant predictors (p < 0.05) were subsequently included in the multivariable analysis. These analyses were re-performed considering repeated consultation in children for the same type of NSPS that the mother had previously consulted for. Poisson regression with robust variance estimator was used to estimate incidence rate ratios (IRR) with 95% CI for the association between exposure to maternal consultations for NSPS and consultation frequency for NSPS in children.

All analyses were performed using IBM SPSS Statistics (version 20.0) [25] and STATA (version 12) [26].

## Results

### Characteristics of children

We included 1437 child-mother pairs. Differences in the baseline characteristics of children exposed and unexposed to maternal consultations for NSPS are presented in Table 2. There were no statistically significant differences between children’s baseline characteristics except for child birth order and maternal history of anxiety/depression disorders. More children exposed to maternal consultations for NSPS were “not first” in birth order (44%) than unexposed children (39%, *p* = 0.029). 37% of exposed and 14% of unexposed children had a history of maternal anxiety/depression disorders (*p* < 0.001).

**Table 2:** **Baseline characteristics of children according to exposure to maternal consultations for NSPS**

**Variable**	**Exposed to maternal visits for NSPS (n = 703) n(%)**	**Unexposed to maternal visits for NSPS (n = 734) n(%)**	***p*** **-value**
**Child age group**			
2-6 years	229 (32.6)	254 (34.6)	0.069
7-11 years	188 (26.7)	158 (21.5)	
12-16 years	286 (40.7)	322 (43.9)	
**Child gender**			
Female	392 (55.8)	398 (54.2)	0.594
Male	311 (44.2)	336 (45.8)	
**Mother age group**			
19-28	94 (13.4)	113 (15.4)	0.470
29-39	305 (43.4)	301 (41.0)	
40-50	269 (38.3)	291 (39.6)	
51-61	35 (5.0)	29 (4.0)	
**Child birth order**			
First	391 (55.6)	451 (61.4)	0.029
Not first	312 (44.4)	283 (38.6)	
**Household members’ count** ^**a**^	4 (1)	3 (1)	0.093
**Number of siblings** ^**a**^	1 (1)	1 (1)	0.254
**IMD 2007quintiles** ^**b**^			
I	139 (19.8)	167 (22.8)	0.161
II	136 (19.3)	144 (19.6)	
III	143 (20.3)	154 (21.0)	
IV	166 (23.6)	135 (18.4)	
V	115 (16.4)	129 (17.6)	
**Maternal history of anxiety or depression**			
No	441 (62.7)	635 (86.5)	<0.001
Yes	262 (37.3)	99 (13.5)	

NSPS - non-specific physical symptoms; ^a^Numbers are Median (Interquartile range); IMD – Index of multiple deprivation; ^b^IMD 2007 scores were missing for 4 of exposed (0.5%) and 5 of unexposed (0.7%) children. Differences between exposed and unexposed children were tested using Mann-Whitney U test for household members’ count and number of siblings, and Chi-squared tests for the remaining variables.

### Proportions of children with repeated and frequent consultations for NSPS

**Figure 1:**
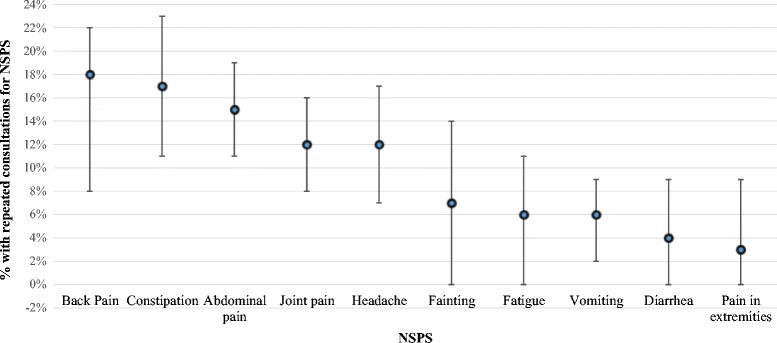
**Percentage of children with repeated consultations for NSPS.** Bars represent percentages with 95% CI; NSPS - non-specific physical symptoms.

27% of the children had repeated consultations for any NSPS. 25% and 17% of all children had repeated consultations for painful and not-painful NSPS, respectively. Repeated consultations for gastrointestinal, musculoskeletal, and neurological symptoms occurred in 15%, 13%, and 10% of children, respectively. The three most common repeated Consultations for NSPS were for back pain (18%), constipation (17%), and abdominal pain (15%); (Figure 1). Consultation frequency for NSPS in children in the two-year period ranged between 1 and 16 consultations (median = 1).

### The associations between maternal consultation for NSPS and repeated NSPS consultations in children

57% of children with repeated consultations for NSPS and 46% of children without repeated consultations for NSPS were exposed to maternal NSPS consultations. Univariable analysis found consultation for any NSPS in mothers was significantly associated with an increased risk of repeated consultation for any NSPS in children (RR 1.41, 95% CI 1.15 to 1.72). This finding remained following adjustment for predictors deemed significant (p < 0.05) in univariable analyses (adjusted RR 1.41, 95% CI 1.16 to 1.73); (Table 3).

**Table 3:** **Risk factors for repeated consultations for NSPS in children**

	**Children with repeated consultations for NSPS (n = 390)**	**Children without repeated consultations for NSPS (n = 1047)**	**Crude RR (95% CIs)**	***P*** **-value**	**Adjusted** ^**a**^ **RR (95% CIs)**	***P*** **-value**
**Maternal visits for any NSPS**						
No	166	568	1		1	
Yes	224	479	1.41 (1.15, 1.72)	0.001	1.41 (1.16, 1.73)	0.001
**Child gender**						
Male	163	484	1			
Female	227	563	1.14 (0.93, 1.40)	0.200	---	---
**Child age group**						
2-6 years	90	393	1		1	
7-11 years	94	252	1.46 (1.09, 1.95)	0.011	1.48 (1.09, 2.01)	0.012
12-16 years	206	402	1.82 (1.42, 2.33)	<0.001	1.89 (1.39, 2.57)	<0.001
**Child birth order**						
Not first	160	435	1			
First	230	612	1.02 (0.83, 1.24)	0.879	---	---
**Household Members’ count** ^**b**^	4 (1)	4 (1)	0.95 (0.88, 1.03)	0.254	---	---
**Number of siblings** ^**b**^	1 (1)	1 (1)	1.00 (0.90, 1.11)	0.945	---	---
**IMD 2007 quintiles**						
I	75	231	1			
II	70	210	1.02 (0.74, 1.41)	0.905	---	---
III	84	213	1.15 (0.85, 1.58)	0.367	---	---
IV	94	207	1.27 (0.94, 1.73)	0.118	---	---
V	65	179	1.09 (0.78, 1.52)	0.623	---	---
**Maternal age group**						
19-28 years	47	160	1		1	
29-39 years	148	458	1.08 (0.78, 1.49)	0.663	0.83 (0.58, 1.18)	0.298
40-50 years	170	390	1.34 (0.97, 1.85)	0.078	0.85 (0.58, 1.25)	0.414
51-61years	25	39	1.72 (1.06, 2.80)	0.028	0.97 (0.57, 1.69)	0.933
**Maternal history of anxiety or depression**						
No	276	800	1			
Yes	114	247	1.23 (0.99, 1.53)	0.062	---	---

NSPS - non-specific physical symptoms; RR - Relative risk; CI - Confidence intervals; IMD – Index of multiple deprivation; ^a^Only child age group, maternal age group, and maternal visits for any NSPS were retained in the final model; ^b^Numbers are Median (Interquartile range).

In addition to maternal consultation for any NSPS, child age was the only statistically significant predictor of repeated consultations for any NSPS; those aged 7-11 and 12-16 years had a one and a half times and almost two times the risk of repeated consultation compared to the youngest age group (RR = 1.48, 95% CI 1.09, 2.01 and 1.89, 95% CI 1.39, 2.57 respectively).

With regards to repeated consultations for more specific types of NSPS, we found significant associations between maternal consultations for painful, gastrointestinal, and neurologic NSPS and repeated consultations for the same type of NSPS in children after adjustment for other significant predictors (child age, maternal age, and maternal history for anxiety/depression; Table 4). Children exposed to maternal consultations for painful NSPS were at 53% increased risk of consulting for painful NSPS as compared to non-exposed children (adjusted RR 1.53, 95% CI 1.18, 1.97).

**Table 4:** **Risk for repeated consultations for specific NSPS in children, by history of maternal consultation for the same specific NSPS**

	**Children with repeated consultations for the same type of NSPS in their mothers**	**Children without repeated consultations for the same type of NSPS in their mothers**	**Crude RR (95% CIs)**	***P*** **-value**	**Adjusted RR (95% CIs)**	***P*** **-value**
**Maternal visits for painful NSPS**						
No	116	443	1		1	
Yes	124	276	1.49 (1.16, 1.92)	0.002	1.53^a^ (1.18, 1.97)	0.001
**Maternal visits for not-painful NSPS**						
No	66	392	1		1	
Yes	23	100	1.30 (0.81, 2.09)	0.282	1.18^b^ (0.72, 1.92)	0.511
**Maternal visits for gastrointestinal NSPS**						
No	81	499	1		1	
Yes	29	92	1.72 (1.12, 2.62)	0.013	1.72^c^ (1.12, 2.62)	0.013
**Maternal visits for musculoskeletal NSPS**						
No	48	260	1		1	
Yes	33	106	1.52 (0.98, 2.37)	0.063	1.49^d^ (0.95, 2.30)	0.084
**Maternal visits for neurological NSPS**						
No	23	175	1		1	
Yes	11	34	2.10 (1.03, 4.32)	0.042	2.10^e^ (1.03, 4.32)	0.042

NSPS - Non-specific physical symptoms; RR - Relative risk; CI - Confidence intervals; ^a^Adjusted for child and maternal age groups; ^b^Adjusted for maternal history for anxiety/depression; ^c^Only maternal visits for gastrointestinal NSPS was retained in the final model; ^d^Adjusted for child age group; ^e^Only maternal visits for neurological NSPS was retained in the final model.

### The associations between maternal consultation for NSPS and frequency of consultation for NSPS in children

**Figure 2:**
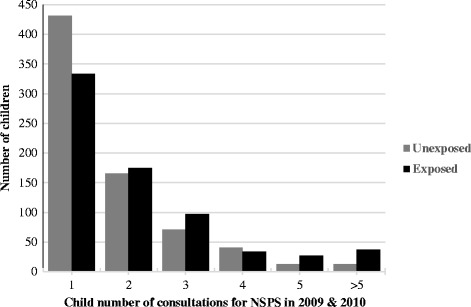
**Number of child’s NSPS consultations by exposure status to maternal consultation for NSPS.**

The median number of consultation for NSPS in the two-year period was 2 (range 1-12) consultations for exposed children and 1 consultation (range 1-16) for unexposed children. Figure 2 shows the number of NSPS consultations in children by exposure status to maternal consultation for NSPS. Maternal consultation for NSPS was associated with a 22% increase in the incidence rate of consultation for NSPS in children (unadjusted IRR 1.22; 95% CI 1.13, 1.31). This association remained significant (IRR 1.21; 95% CI 1.12, 1.31) after adjusting for other significant predictors (child age group, maternal age group, and maternal history of anxiety/depression).

## Discussion

This study found that over a quarter (27%) of children had repeated consultations for NSPS. The most common repeated consultations for NSPS in children were for back pain, constipation, and abdominal pain. We found significant associations between consultations for different types of NSPS in mothers and repetition of similar consultations in their children. These associations were clearest for any NSPS, painful, gastrointestinal, and neurological symptoms. We also found that exposure to maternal consultation for NSPS was associated with increase in consultation frequency for NSPS among children.

### Comparison with other studies

Our findings are similar to those reported in previous studies. A systematic review examining the recurrence/persistence of abdominal pain among children reported that 29% of children with baseline abdominal pain had recurrent/persistent abdominal pain using various follow-up periods, ranging between 1-5year periods [27]. Previous studies also reported that children of parents with irritable bowel syndrome (IBS) have more physician consultations for NSPS than children of parents without IBS [28,29]. This is the first cohort study to examine repeated physician consultations for NSPS in children, in relation to maternal physician consultations for NSPS. Prior research, using self-reported data, provides indirect evidence to support the findings of the current study. One study found that NSPS among adults aged 36 years were significantly associated with abdominal pain and poor parental health when participants were aged 15 years [30]. In the same study, headache in childhood was linked to headache (odds ratio (OR) 2.22, 95% CI 1.62 to 3.06) and multiple NSPS (OR 2.22, 95% CI 1.62 to 3.06) in adulthood.

### Interpretation

The exact mechanisms underlying the associations between maternal consultation for NSPS and repetition of similar and frequent consultations in children are still to be determined. Research evidence from primary care and population-based studies suggest that genetic, social and environmental factors may be involved. There is some evidence that genetic effects contribute to the onset of some NSPS, including headache and IBS [31-33]. However, it seems unlikely that genetic factors are able to fully explain this because the observed associations were only significant for painful NSPS, and repeated consultations for NSPS increased with increasing child’s age. Research suggests that exposure of family members to certain social and environmental factors (e.g. socioeconomic circumstances and poor family functioning) is associated with greater reporting of NSPS [34,35]. Another plausible explanation for our findings is childhood social learning of illness behavior (such as family definition of illness, recognition and perceived seriousness of symptoms, and reaction to symptoms), which has been hypothesized to play an important role in the development of illness and health-seeking behavior in children [4,36]. A number of studies have suggested that parental responses and attitudes toward the child’s illness (reinforcement) and parental coping mechanisms with their own illness (role modelling) may influence symptoms frequency, disability days, and healthcare consultations in their children when they become adults [37,38]. For example, women with IBS were more likely than women without IBS to emulate illness behavior of their parents and to recall that their parents reinforced their illness behavior by rewarding them with special privileges [37].

### Strengths and limitations

This study is strengthened by using documented physician consultations and prospective data collection. The child’s exposure to maternal consultations was measured using electronic medical records, which is considered a more reliable source of data than recall [39]. Another important strength is that the exposure to maternal consultations for NSPS was ascertained before studying the repetition of consultations for NSPS in children, which provides a clear temporal relationship. Additionally, the CiPCA database has been shown to be a high quality dataset [20].

This study also has some limitations which should be considered. We could not assemble an inception cohort of children presenting for the first time with NSPS, because we have no information on time of onset of these symptoms. Additionally, selecting those with new episodes of healthcare consultation does not necessarily define the onset of the symptoms, and is therefore of limited value [40]. However, this cohort included a group of consecutive children presenting with NSPS, which was clearly defined and assembled at the time of the child consultation for NSPS. Another potential limitation is diagnostic misclassification, which is a common problem in primary care [41]. However, diagnostic misclassification is unlikely to completely explain our findings due to the high quality of coded clinical data within CiPCA practices. For example, 97% of all physician consultations that occurred in the CiPCA practices in 2006 were given a morbidity code [42]. Additionally, the current classification system used in primary care allows for coding definitive diagnoses (e.g. urinary tract infection) as well as symptom diagnoses (e.g. abdominal pain) when a definitive diagnosis is not established, which reduces the potential for diagnostic misclassification. Another limitation is that because we excluded NSPS consultations in pregnant mothers, children whose mothers were pregnant during the study period may have been less likely to be exposed, especially if younger children were more likely to have mothers who were pregnant. However, this is unlikely to have resulted in systematic bias because the proportions of exposed and unexposed children were comparable in each age group. Additionally, we did not examine whether other alternative diagnoses of well-defined conditions were made for NSPS among children at a later stage because consultation data since 2010 was not available to us when we conducted the study.

### Generalizability

In the UK over 97% of the population is registered with a primary care practice, which usually provides first point access to non-emergency healthcare [43]. This cohort consisted of all children that could be paired with a mother from 12 primary care practices, reducing the chance of selection bias. We also assembled a clearly defined cohort of consecutive consulters for NSPS at the time of their consultation. This enhances the internal and external validity of this study, and therefore these findings are highly likely to be generalizable.

### Implications for clinical practice and future research

This study has provided new prospective evidence that repeated and frequent consultations for NSPS among children are influenced by consultations for NSPS in their mothers. These findings suggest that the potential for repeated and frequent consultations for NSPS in children should be viewed with context of the family. More research is required to fully explain the exact mechanisms underlying the associations between exposure to maternal consultations for NSPS and the frequency and repeated consultations for NSPS in the child. Such research may shed light on effective management strategies to reduce the frequency and the number of consultations for NSPS in children.

## Conclusions

This study showed that considerable proportions of children have repeated physician consultations for NSPS, and that repeated and frequent consultations for NSPS are influenced by previous exposure to maternal consultations for NSPS. Medical practitioners managing children presenting with NSPS in both primary and secondary care should be aware of these links. More research with longer follow-up periods is needed to fully explain the influence of parental health on the health and consulting behavior of children.
